# Sleep

**Published:** 1892-09

**Authors:** 


### SLEEP.

The crying need of American women, says a physician, whose specialty of the nervous diseases brings him in contact with plenty of the nervous type of the sex, is sleep. Over and over I tell my women patients : sleep all you can, nine, ten hours every night, and, no matter how much at night, sleep surely one hour of daylight. Many of them reply : I don't have time to sleep during the day. Take time, say I ; you'll get it back, good measure, pressed down, running over. Then they can't sleep in the day-time. That is nonsense. They may not the first few days, but very soon, after persistently making the effort every day, at a certain time, the habit will be formed.

